# A Novel Vibration Piezoelectric Generator Based on Flexible Piezoelectric Film Composed of PZT and PI Layer

**DOI:** 10.3390/polym14142871

**Published:** 2022-07-15

**Authors:** Jia Wang, Yujian Tong, Chong Li, Zhiguang Zhang, Jiang Shao

**Affiliations:** School of Mechanical Engineering, Jiangsu University of Science and Technology, Zhenjiang 212100, China; wjjzhb@just.edu.cn (J.W.); 202020030@stu.just.edu.cn (Y.T.); 172210202331@stu.just.edu.cn (Z.Z.)

**Keywords:** piezoelectric generator, PI film, experimental analysis, simulation

## Abstract

A novel piezoelectric generator based on soft piezoelectric film consisting of a polyimide (PI) sheet and lead zirconate titanate (PZT) is proposed to generate electric energy under the operating conditions of low-frequency and small-amplitude vibration. The theoretical model and working principle of the piezoelectric generator are discussed in detail. Using ANSYS software, a finite element analysis of the static and modal characteristics of the piezoelectric generator is carried out. Further, the output of the prepared piezoelectric generator is investigated by a home-made experimental platform. Results show that the transient excitation voltage of the generator increases with the increase in load resistance, and the continuous excitation voltage increases first and then remains almost stable. The maximum continuous power produced by the piezoelectric generator is about 4.82 mW. Furthermore, the continuous excitation voltage and power are in accordance with the simulation values when the load resistances are 20 kΩ and 25 kΩ, respectively.

## 1. Introduction

Recently, renewable energies have gained considerable attention due to the negative effects of fossil fuel consumption, such as global warming [1,2]. As a result, significant efforts have been devoted to developing advanced energy-harvesting technologies to generate electric power from renewable energies, such as solar, ocean wave, wind, mechanical vibration and so on [3,4].

Among these renewable energies, mechanical vibration is an attractive option because of its abundance in the natural environment [5]. Moreover, mechanical-vibration-based generator has the advantages of relative high-power density, higher potential and longer lifespan [6,7,8,9]. Consequently, several strategies, such as piezoelectric mechanism, electromagnetic mechanism and electrostatic mechanism, have been introduced to develop a mechanical-vibration-based energy harvester [10,11,12,13]. The piezoelectric generator is based on the piezoelectric mechanism, which has been extensively investigated and is understood to be related to mechanical–electrical energy conversion. For instance, using the longitudinal vibration of the drill pipes, Zheng et al. [14] designed a while-drilling energy-harvesting device. The designed energy-harvesting device was utilized as a continuous power supply for downhole instruments during the drilling procedure. When the thickness of the piezoelectric patches was 1.2–1.4 mm, the designed device presented the best energy harvest performance, with a peak voltage of 15–40 V. To monitor the random vibration of rails, an efficient rail-borne piezoelectric energy harvester was used to collect energy from the random railway vibrations by Yang et al. [15]. The output power peaks at the first two resonance frequencies were 1036.9 and 8.01 mW/Hz. To improve the working frequency bandwidth and environmental robustness of the piezoelectric vibration energy harvester, a multi-frequency response piecewise-linear piezoelectric vibration energy harvester was developed [16]. Based on the electromechanical coupling and the dynamic response, a theoretical model of the energy harvester was established. Results showed that the energy generated by the multi-frequency response piecewise-linear piezoelectric energy harvester was 194% of the energy generated by its linear counterpart under the same excitation conditions. Vibration energy harvesting from backpacks has the potential to generate electrical power, and various energy-harvesting backpacks have been designed. However, dynamics between the human body and the backpack have an important influence on the dynamic performance of the backpacks. Therefore, to improve human comfort, Liu et al. [17] investigated the dynamic interaction between the human body and the energy-harvesting backpacks. It was found that tuning the backpack parameters can reduce the ground reaction forces in the push-off phase and potentially improve human comfort. To overcome the low energy utilization of a traditional piezoelectric energy harvester, a uniform stress distribution of bimorph has been designed for piezoelectric energy harvesting [18]. Further, Morel et al. [19] proposes a general, normalized, and unified performance evaluation of the various electrical strategies to tune the harvester’s frequency response. With a thorough analysis, the influence of the tunable electrical interfaces on the electromechanical generator response was investigated.

As the key components of the piezoelectric generator, piezoelectric smart materials, typically PZT, possessing advantages of small size, fast response and high energy density are widely utilized in energy-harvesting applications [20,21,22], and a number of PZT-based generators have been developed. A piezoelectric generator based on vortex-induced vibration was proposed to convert flow energy underwater to electrical energy [23]. This generator consists of a piezoelectric cantilever beam, connecting device, springs, bluff body and displacement sensor. The output voltage is derived from the flow–solid-electric coupling equations. The experimental test of the piezoelectric generator performance at different water speeds shows good agreement with the theoretical results. Due to the inefficiency of harvesting energy from low-frequency vibrations of traditional piezoelectric cantilever structures, a novel piezoelectric generator used a cantilevered bimorph with a tip mass and a pair of preloading springs was designed [24]. The harvester was modeled as a Euler–Bernoulli beam, and the piezoelectric material is assumed to be linear. It was found that changing the preloading of the spring helped reduce the natural frequency of the cantilever. Further, in order to improve the adaptive range of airflow velocity, an airflow piezoelectric generator based on multi-harmonic excitation was proposed [25]. The flow field characteristics were obtained by the computational fluid dynamics (CFD) method. The result showed that periodic compression and expansion of air were presented and a standing wave was formed inside the resonator. The results verified the viability of the multi-harmonic excitation energy-exchange method. An eye-shaped generator consisting of a rectangular ceramic and two elastic body plates was also developed [26]. Once tension is applied to both ends of the elastic body, it causes the elastic body to deform, resulting in a positive piezoelectric effect to generate electric energy. The proposed generator is relatively durable because the forces are not applied directly to the ceramic. However, despite their outstanding piezoelectric properties, ceramic materials are limited in the field of energy collection owing to their inherent disadvantages, such as rigidity, brittleness and low voltage coefficient [27,28,29].

Therefore, piezoelectric polymers were introduced to overcome the abovementioned drawbacks of ceramic materials. Piezoelectric polymers demonstrate better dielectric behavior and field strength, being able to tolerate high driving voltage [30]. The popular piezoelectric polymers are polyvinylidene fluoride (PVDF), polylactic acids (PLA), polyurethanes (PU) and PI [31,32,33,34]. As a promising piezoelectric material, PI has been widely used, owing to its outstanding thermal stability, its excellent mechanical durability and the exceptional designability of its piezoelectric properties. For instance, Dagdeviren et al. [35] reported a piezoelectric generator to collect mechanical energy generated from the movement of heart, lung, and diaphragm. The developed piezoelectric generator was based on PZT and PI film, where PI worked as flexible matrix and encapsulation layer, while PZT was in a configuration of ribbons sandwiched between gold and platinum electrodes. The averaged power density of the proposed piezoelectric generator reached 1.2 μW/cm^2^.

Although significant progress has been made, it still remains a challenge to gather electric energy under small-vibration amplitude. In this paper, a novel vibration piezoelectric generator capable of amplifying the vibration amplitude and generating electrical energy was developed based on soft piezoelectric PZT/PI film. The performance of prepared generator was evaluated by a home-made experimental platform. The results indicated that the maximum output of the developed piezoelectric generator could reach 4.82 mW. Moreover, simulation studies with respect to continuous excitation voltage and power were also conducted, and showed good agreement with experimental results, while the load resistances were chosen to be 20 kΩ and 25 kΩ, respectively.

## 2. Operating Principle

In a typical cantilever beam structure piezoelectric generator, one end of the cantilever beam is fixed, and the other end mechanically vibrates [36,37,38,39], transforming mechanical energy into elastic potential energy. The alternating voltage is generated by releasing the strained piezoelectric layer.

**Figure 1:**
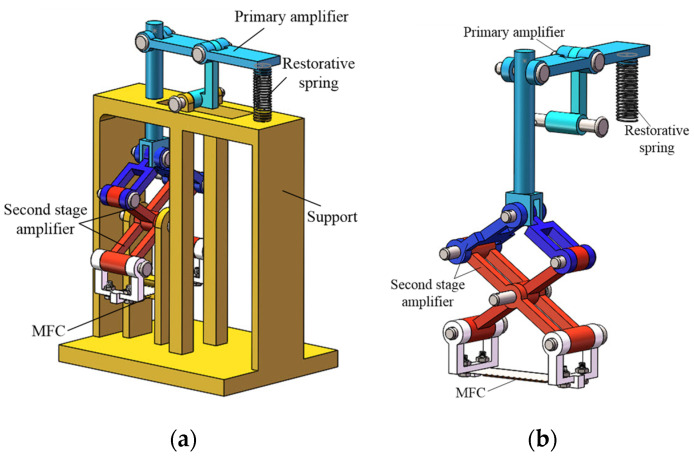
Structure of the vibration piezoelectric generator. (**a**) Overall structure; (**b**) amplitude amplification mechanism.

In this study, the proposed piezoelectric generator 2qs based on a two-stage amplitude amplification mechanism, as shown in Figure 1. In the piezoelectric power generator, the amplification mechanism 2qs added to the force transfer mechanism, which was used to evenly transfer the force to both ends of the elastic mechanism, so that both ends of the elastic mechanism could achieve the same bending, and the bending degree was the same. The piezoelectric generator consisted of piezoelectric macro fiber composite (MFC), primary amplifier, second stage amplifier, restorative spring and support. The primary amplifier was responsible for the first step amplification of vibration amplitude by means of lever amplification principle, whereas the second amplifier achieved the second amplification of the amplitude by using an X–type mechanism. The piezoelectric MFC was attached to an elastic steel plate and deformed simultaneously with the elastic steel plate. The restorative spring was employed to restore the initial state under the restorative force when the lever of the primary amplifier was pressed.

**Figure 2:**
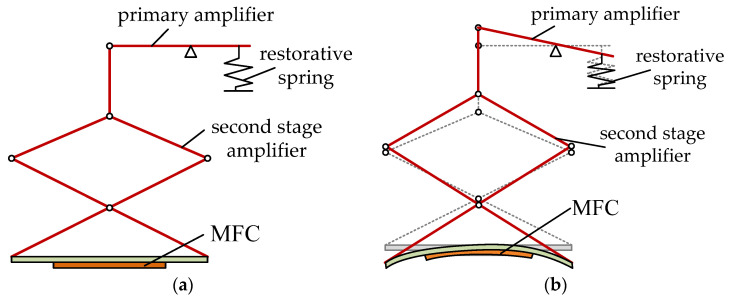
Operating principle of the vibration piezoelectric generator. (**a**) The MFC is not deformed; (**b**) the MFC is deformed.

The operating principle of the proposed vibration piezoelectric generator is shown in Figure 2. At the initial moment, the primary amplifier is in the horizontal state and the second stage amplifier is in a relaxed state with undeformed MFC. In this state, the restorative spring is free. When the vibration acts on the end of the primary amplifier, the restorative force spring at the end of the lever is compressed. Under the action of the primary lever, the secondary amplifying mechanism is lifted to drive the bending deformation of both ends of the MFC. Due to the positive piezoelectric effect, the bending deformation of MFC produces voltage. Under the cyclic vibration, continuous voltage output is obtained at both ends of the MFC.

Compared with other kinds of piezoelectric generators [36,37,38,39], the proposed piezoelectric generator has the function of multistage displacement amplification. The primary amplification mechanism realizes the first amplification of vibration displacement through the lever principle, and the second-stage amplification mechanism can realize multistage amplification of vibration displacement. Therefore, even if the external input vibration displacement is very small, the piezoelectric generator can also generate large electric energy through the displacement amplification function.

**Figure 3:**
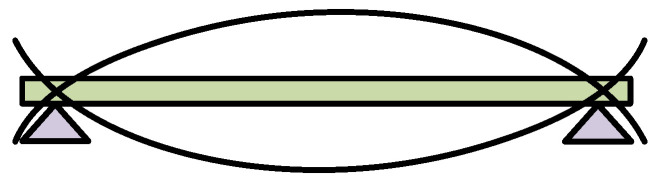
Fixed mode of the elastic steel plate.

Here, the fixing mode at both ends of the elastic steel plate can be simplified into simple support at both ends, as shown in Figure 3. When the piezoelectric generator is in operation, the elastic steel plate can be regarded as consisting of two cantilever plates. When external vibration is applied to the input end of the piezoelectric generator, the vibration displacement is amplified by multistage displacement amplification. Multistage displacement amplification mechanism drives the reciprocating motion at both ends of the elastic steel plate. Under multiple actions, the piezoelectric MFC produces bending deformation, which in turn generates electrical energy under the piezoelectric positive effect.

## 3. Electromechanical Model

**Figure 4:**
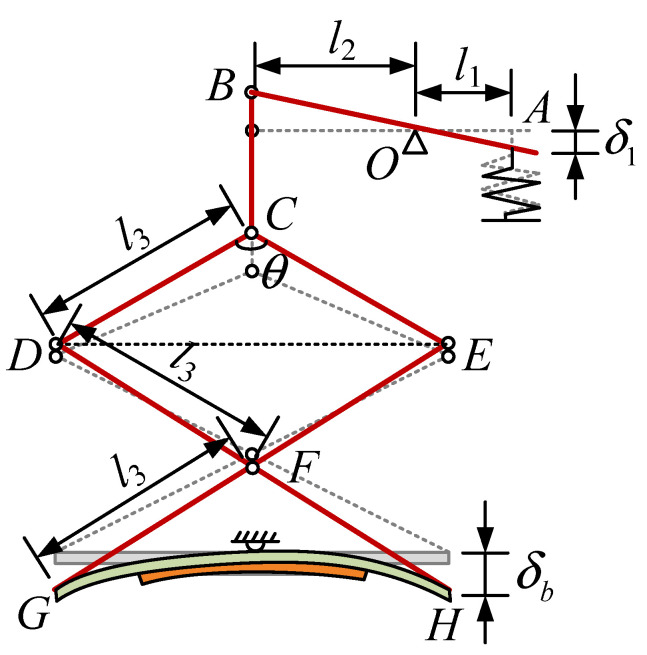
Displacement calculation diagram of each part under external excitation.

The proposed vibration piezoelectric generator amplifies the amplitude through two-stage amplification, and the efficiency of energy collection is improved. Figure 4 presents the displacement calculation diagram of each part under external excitation, where *δ*_1_ is excitation amplitude value.

For the proposed piezoelectric generator, a sinusoidal excitation of amplitude *δ*_1_ is used, and it is expressed as:

(1) $$e\left(t\right)=\delta_{1}\sin\left(2\pi ft\right),$$

where *f* is exciting frequency. Under the action of primary amplifier, the displacement amplitude of the upper end of the second-stage amplification is calculated as:

(2) $$\delta_{2}\left(t\right)=\frac{l_{2}\delta_{1}}{l_{1}}\sin\left(2\pi ft\right),$$

where *l*_1_ and *l*_2_ are the lengths of the two sections of the lever. The second magnification consists of three isosceles triangles, and the angle between the two equal sides is *θ*. With the first lever amplification, the triangle *CDE* is elevated by *δ*_2_. Then the angle between equal sides is expressed as:

(3) $$\theta_{1}\left(t\right)=2\arccos\left(\cos\frac{\theta }{2}+\frac{\delta_{2}\left(t\right)}{l_{3}}\right),$$

where *l*_3_ is equilateral length of a triangle. The height increment of Δ*DFE* and Δ*GFH* can be written as:

(4) $$\delta_{3}\left(t\right)=l_{3}\left(\cos\frac{\theta_{1}\left(t\right)}{2}-\cos\frac{\theta }{2}\right)$$

The displacement at the end of the MFC base is denoted as:

(5) $$\delta_{b}\left(t\right)=2\delta_{3}\left(t\right)+\delta_{2}\left(t\right)-\delta_{2}\left(t\right)=2\delta_{3}\left(t\right),$$

The length of the MFC base and MFC are *l_b_* and *l_p_*, respectively. Therefore, the displacement amplitude at the end of the piezoelectric MFC is calculated as:

(6) $$\delta_{p}\left(t\right)=\delta_{b}\left(t\right)\frac{l_{p}}{l_{b}},$$

**Figure 5:**
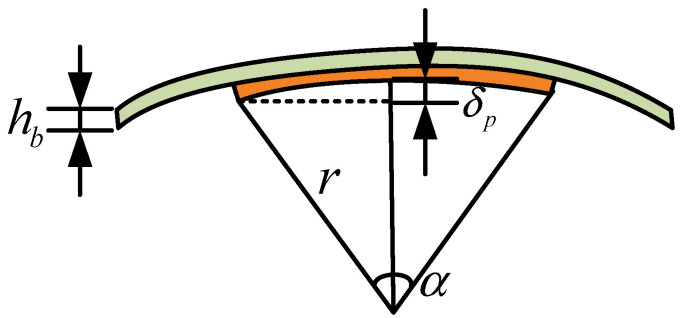
MFC deformation calculation diagram.

Figure 5 shows the deformed MFC, where *r* and *α* are the radius and angle of the deformed MFC, respectively. The relationship between *r* and *α* can be derived as:

(7) $$\left\{ \begin{array}{l} \alpha r=l_{p}\\ r\left(1-\cos\frac{\alpha }{2}\right)=\delta_{p} \end{array}\right.,$$

From Equation (7), it can be concluded that

(8) $$\alpha =\frac{l_{p}}{\delta_{p}}\left(1-\cos\frac{\alpha }{2}\right),$$

Therefore, the shortened length of MFC is expressed as:

(9) $$\begin{array}{l} \delta_{M}\left(t\right)=\alpha \left(r+h_{b}\right)-\alpha r\\ \;\;\;\;\;\;\;=\frac{l_{p}h_{b}}{\delta_{p}\left(t\right)}\left(1-\cos\frac{\alpha }{2}\right) \end{array},$$

where *h_b_* is the thickness of the MFC base.

**Figure 6:**
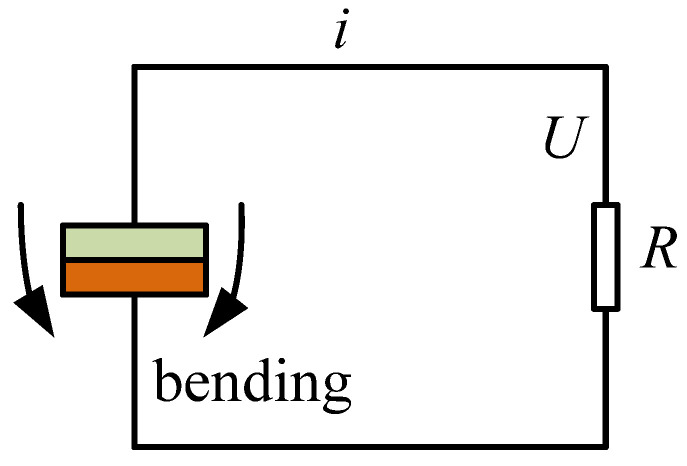
Equivalent circuit of piezoelectric MFC.

Figure 6 presents the equivalent circuit of piezoelectric MFC, where *R* is load resistance. When the piezoelectric MFC is subjected to bending deformation, assuming a linear change between stress and strain, the output charge is [40]

(10) $$Q\left(t\right)=d_{31}ES_{p}\frac{\delta_{M}\left(t\right)}{l_{p}},$$

where *d*_31_, *E*, *S_p_* are piezoelectric strain constant, Young modulus of MFC and surface area of the piezoelectric material, respectively.

The output current of load is calculated by:

(11) $$i\left(t\right)=\frac{\partial Q\left(t\right)}{\partial t}=d_{31}ES_{p}\frac{\partial \delta_{M}\left(t\right)}{l_{p}\partial t},$$

The output power of the proposed piezoelectric generator is expressed as:

(12) $$P\left(t\right)=\frac{Q^{2}\left(t\right)}{2C}=d_{31}^{2}E^{2}S_{p}^{2}\frac{\delta_{M}^{2}\left(t\right)}{2Cl_{p}^{2}},$$

where *C* is the capacitance of piezoelectric MFC. The effective value of the output voltage is calculated by the following expression:

(13) $$U_{eff}\left(t\right)=\sqrt{P\left(t\right)R},$$

Thus, the peak output voltage of the generator can be derived as:

(14) $$U_{p-p}\left(t\right)=\sqrt{2}U_{eff}\left(t\right),$$

## 4. Structural Simulation Analysis

In order to verify the rationality of the designed structure, the finite element simulation software ANSYS was used to simulate the structural performance of the proposed piezoelectric generator. The elastic steel plate of the piezoelectric vibrator is 65 Mn-high elastic quenched spring steel plate with a thickness of 0.1 mm. Due to its excellent production process, the 65 Mn-high elastic quenched steel plate has the characteristics of high elasticity, high wear resistance, high toughness and good flatness. Its mechanical properties were good, with high tensile strength and elongation. Therefore, it is suitable as an elastic plate for the piezoelectric vibrator in a vibratory piezoelectric energy-collecting device.

The support structure and the force transfer mechanism in the device are made of PLA material and 3D-printed in a single form. The printed structure has the characteristics of high cleanliness and strength, fine appearance, etc. In addition, the accuracy also meets the requirements, being suitable for the assembly verification of the experimental device and for the appearance and structural components of this design. The printed device met the requirements after assembly.

Before the ANSYS simulation, it was necessary to set the basic parameters of the material of the piezoelectric generator, including the material density, elastic modulus and Poisson’s ratio. Only by setting these parameters accurately could the desired effect be achieved in the simulation and the correct results obtained. Table 1 shows the basic parameters of materials, while Table 2 gives the parameter information of the MFC piezoelectric film. The piezoelectric MFC film was composed of a piezoelectric material with two sides, and an adhesive backing sheet attached on one side. The slicing of the piezoelectric material constituted a plurality of piezoelectric fibers in juxtaposition. A conductive film was then adhesively bonded to the other side of the piezoelectric material, and the adhesive backing sheet was removed. The conductive film had first and second conductive patterns formed thereon, which were electrically isolated from one another and in electrical contact with the piezoelectric material. The first and second conductive patterns of the conductive film each had a plurality of electrodes forming a pattern of interdigitated electrodes. A second film was then bonded to the other side of the piezoelectric material. The second film may have had a pair of conductive patterns similar to the conductive patterns of the first film [41].

Finite element analysis can simplify complex problems and obtain an appropriate and similar solution for each small problem. In the working process of the proposed piezoelectric generator, the components of the generator re subjected to more or less tension, pressure or bending deformation, so the statics analysis of the device could be carried out using ANSYS finite element analysis software to analyze its overall deformation and stress.

**Figure 7:**
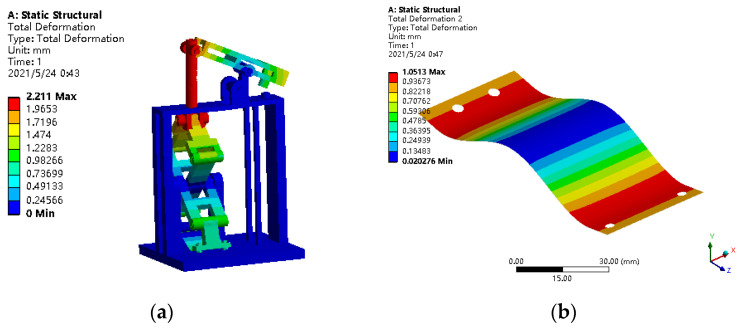

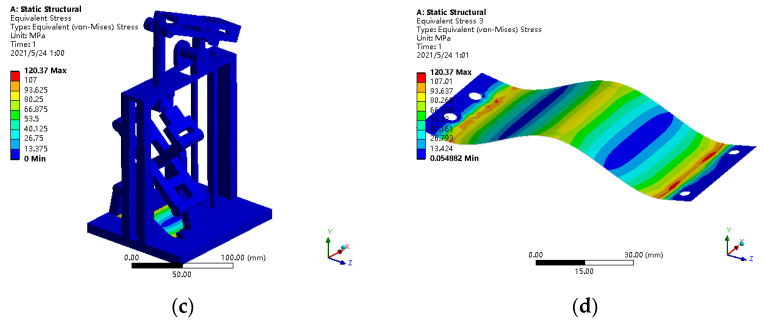
Deformation and stress of piezoelectric generator under external excitation. (**a**) Overall deformation; (**b**) elastic steel plate deformation; (**c**) overall stress; (**d**) elastic steel plate stress.

The static analysis of vibratory piezoelectric generator was carried out using ANSYS software. In order to verify the performance of the piezoelectric generator, the structural deformation of the piezoelectric generator under low amplitude excitation was simulated, and the amplitude of external excitation was set as 0.5 mm to solve the deformation of each part of the piezoelectric generator. At the same time, in order to study the characteristic of piezoelectric generator under external excitation, the stress of the piezoelectric generator was analyzed. The deformation and stress analysis results are shown in Figure 7.

As shown in Figure 7a,b, the overall maximum deformation of the piezoelectric generator was 2 mm, and the maximum deformation was located at the vertical rod of the piezoelectric generator, which met the allowable requirements. Other connectors of the generator also had more or less deformation under the action of external forces, but they were less than 2 mm, meeting the use requirements for PLA materials. Moreover, the maximum deformation of the elastic steel plate, the key component of the piezoelectric generator, was only 1 mm.

According to the mechanical properties of the material, the tensile and compression yield strength of PLA was 40–51 MPa, and the yield limit of the elastic steel plate was 825–925 MPa. In the finite element analysis results of Figure 7c,d, the maximum stress of the elastic steel plate was 120.37 MPa, less than the yield strength of the spring steel. In addition, the overall stress of the external structure of the piezoelectric generator was less than 14 MPa, so it met the design requirements.

**Figure 8:**
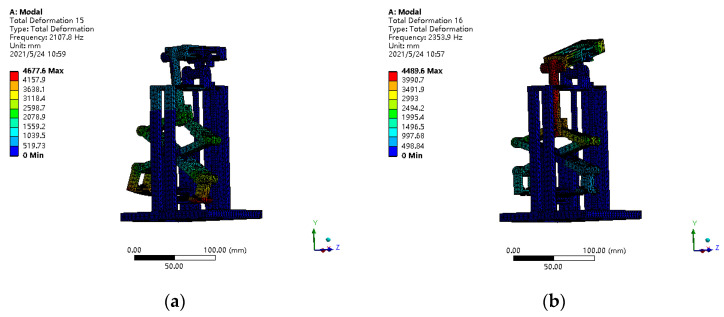

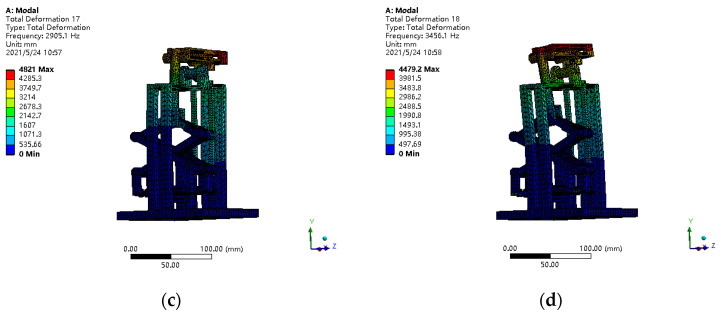
Modal shapes of piezoelectric generator. (**a**) First-order modal shape; (**b**) second-order modal shape; (**c**) third-order modal shape; (**d**) fourth-order modal shape.

The resonance of the structure is destructive to the proposed piezoelectric generator. Therefore, in order to avoid the resonance of the piezoelectric generator in the working process, the excitation frequency should be far away from the resonance frequency. Therefore, modal characteristics of piezoelectric generator were analyzed in this paper. Table 3 shows the natural frequencies of the piezoelectric generator, and Figure 8 presents the modal shapes of the piezoelectric generator.

According to Figure 8, the first-order natural frequency of the piezoelectric is 2107.8 Hz, and the main vibration mode is the bending vibration of the elastic steel plate. At the same time, with the increase in the frequency order, the main mode shape changes to the displacement amplification mechanism.

Since the piezoelectric generator designed in this paper mainly works in the low-frequency range less than 100 Hz, it does not generate resonance during the working process.

## 5. Experimental Analysis

**Figure 9:**
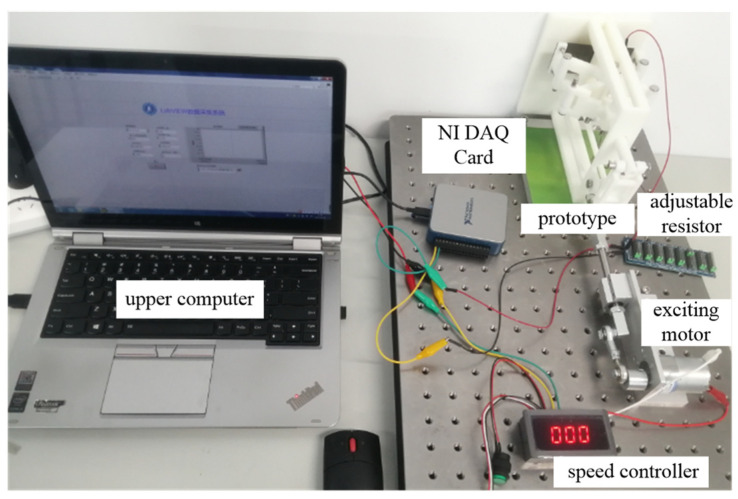
Experimental test system of the proposed piezoelectric generator.

In order to test the performance of the piezoelectric generator, an experimental platform was manufactured, as shown in Figure 9. The generator was manufactured using 3D printing technology. The vibration excitation of the generator was realized by the reciprocating motion of a DC motor, and a speed controller was used to adjust the excitation frequency. An adjustable resistor varying in the range of 0–10 MΩ was utilized as load resistor. The output voltage of the developed generator was collected by an NI USB-6002 data acquisition (DAQ) card. The upper computer installed with LabVIEW acquisition software was used to analyze the test data.

**Figure 10:**
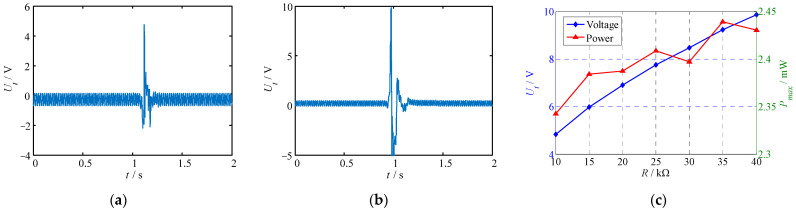
Instantaneous voltage and power of the proposed piezoelectric generator. (**a**) Instantaneous voltage at *R* = 10 KΩ; (**b**) instantaneous voltage at *R* = 40 KΩ; (**c**) instantaneous power.

Firstly, the voltage generated by the piezoelectric generator under instantaneous excitation was evaluated, as illustrated in Figure 10a,b. Here, through changing the load resistance, the instantaneous response law of the output voltage was obtained. Figure 10c shows the instantaneous maximum voltage and maximum power of the generator vary with load resistance. Results show that:

(1) Under instantaneous vibration excitation, the generator generated transient voltage response, and the response time lasted 0.2 s–0.3 s. Under different load resistances, the voltage response curves of the piezoelectric generator were similar, and the response amplitudes were different. When the load resistance increased, the maximum voltage response amplitude also increased.
(2) As the load resistance *R* increased, the maximum output voltage increased. For the output power, the maximum instantaneous power occurred at *R* = 35 KΩ and was 2.44 mW.

**Figure 11:**
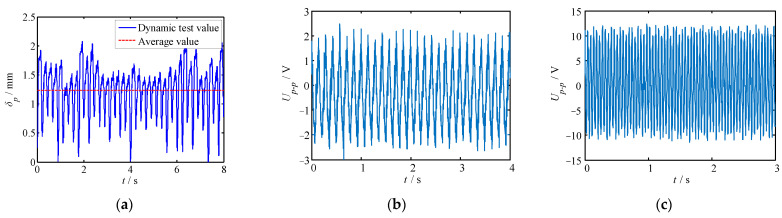
MFC displacement and continuous voltage response of the piezoelectric generator. (**a**) MFC displacement; (**b**) continuous voltage at *R* = 10 kΩ, *f* = 10 Hz; (**c**) continuous voltage at *R* = 40 kΩ, *f* = 25 Hz.

The voltage response of the piezoelectric generator under continuous excitation was also tested. Using a laser displacement sensor with a range of ±5 mm, the vibration amplitude of the piezoelectric MFC was measured, and the test result is shown in Figure 11a. Under continuous vibration excitation, the piezoelectric MFC generated a continuous voltage signal. The images in Figure 11b,c show the voltage-response test results of the piezoelectric generator under continuous excitation with different frequencies and different load resistances. According to Figure 11, it can be noted that:

(1) Under the excitation of a continuous vibration signal, the displacement of the piezoelectric MFC changed regularly and continuously in the range of 0–2 mm. The average displacement was 1.23 mm.
(2) At different exciting frequencies and load resistances, the voltage response of the piezoelectric generator was similar. The difference was that the maximum output voltage increased with the increase in excitation frequency and load resistance.
(3) When the excitation frequency and load resistance were constant, the output voltage of the generator varied regularly with time. The output voltage waveform approximated sinusoidal law.

**Figure 12:**
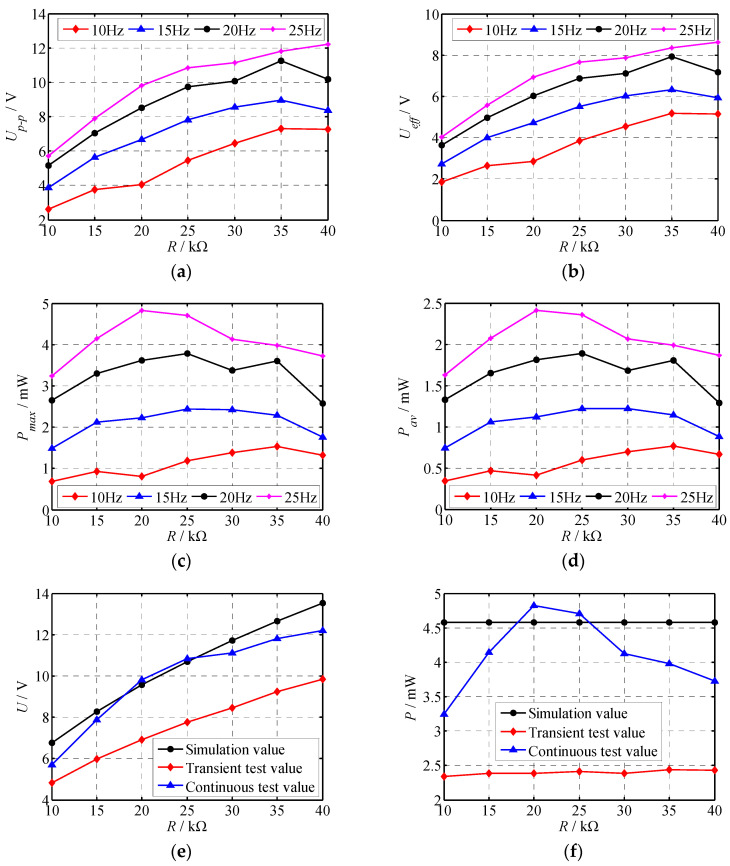
The output voltage and power vary with the load resistance. (**a**) Maximum voltage; (**b**) effective voltage; (**c**) maximum power; (**d**) average power; (**e**) comparison of experimental and simulated voltages; (**f**) Comparison of experimental and simulated powers.

The variation in voltage with load resistance at different frequencies was tested experimentally. From the relationship between voltage and power, the variation of power with load resistance was obtained. The images in Figure 12a,b show the output voltage variations with the load resistance. The images in Figure 12c,d show the output power variations with the load resistance. Additionally, the electric generating test results under instantaneous excitation and continuous excitation were compared with the respective simulation results. The comparison results are shown in Figure 12e,f. From Figure 12, it can be observed:

(1) With the increase in load resistance, the peak voltage and effective value first increased, and then tended to be stable or even slightly decrease. The maximum peak voltage and effective voltage were 12.22 V and 8.64 V, respectively, and occurred at the load resistance of 40 kΩ and the excitation frequency of 25 Hz. When the load resistance was constant, the peak voltage and effective voltage increased with the increase in excitation frequency. When the load resistance was 40 kΩ, the voltage change was more obvious.
(2) When the excitation frequency was less than 20 Hz, the maximum power and average power tended to be stable with the change in load resistance. When the excitation frequency was greater than 20 Hz, the output power was not stable. The power decreased with the increase in load resistance. The maximum power and average power of the piezoelectric generator were 4.82 mW and 2.41 mW, respectively, occurring at the load resistance of 20 kΩ and the excitation frequency of 25 Hz.
(3) The instantaneous voltage of the generator was very close to the simulated voltage. The maximum error between the instantaneous voltage corresponding to different load resistor and the simulation voltage was 15.7%, which occurred when *R* was 10 kΩ. The instantaneous voltage and simulation voltage had the same variation rule, and the instantaneous voltage was less than the continuous voltage.
(4) As the load resistance changed, the maximum simulation power values remained unchanged. The reason was that the output power was not affected by the load resistance based on Equation (12). The instantaneous power fluctuated within the range of 2.34 mW–2.44 mW. The continuous power increased first and then decreased, and its power range was 3.25 mW–4.82 mW. When the load resistance *R* was 20 kΩ and 25 kΩ, the continuous power was close to the average power, and the errors were 5.24% and 2.84%, respectively. There was a significant difference between instantaneous power and continuous power. For instantaneous power, which was generated only for a very short time, due to the lack of continuous excitation, its power was relatively small. For continuous power, the maximum output power was close to the calculated value. When the load resistance was small, the energy was not fully released and the power was small, while when the load resistance was large, the stability of the output power was reduced and the power was slightly reduced. Hence, the instantaneous power and continuous power in Figure 12f were inconsistent with the calculated results.

Therefore, the output power of the piezoelectric generator under continuous excitation is closer to the simulated power, especially under certain loads. When the load resistance is 20 kΩ, the generator shows better performance.

## 6. Conclusions

In this paper, a low-frequency vibration-type piezoelectric generator based on the PZT-PI composite film was proposed. The theoretical model and working principle of the piezoelectric generator were presented and discussed. In addition, a home-made experimental platform was built to test the output characteristics of the piezoelectric generator. Results show that:

(1) The transient excitation voltage of the proposed piezoelectric generator increased with the increase in load resistance, while the continuous excitation voltage increased first and then tended to be stable or slightly decreased.
(2) The maximum continuous power produced by the piezoelectric generator was about 4.82 mW.
(3) The simulation results agreed well with the experimental results on continuous excitation voltage and power with load resistances of 20 kΩ and 25 kΩ.

## Figures and Tables

**Table 1:** Basic parameters of materials.

Materials	PLA	Spring Steels
Use	Support structure	Elastic vibrator
Density (kg/m^3^)	1.12	7.81
Young’s modius (GPa)	2.7	197
Poisson’s ratio	0.39	0.25
Buk modulus (GPa)	4.1	131
Shear modulus (GPa)	0.97	78.8

**Table 2:** Parameter information of MFC piezoelectric film.

Parameter	Values	Parameter	Values
Working mode	d_31_	Effective working length	28 mm
Thickness	300 μm	Effective working width	14 mm
Electrode	Standard lead-free solder S-Sn99Cu1	Total length	37 mm
Capacitance	48 nF	Total width	18 mm
Upper limit of operating frequency	<1 MHz		

**Table 3:** Natural frequencies of the piezoelectric generator.

Order	First Order	Second Order	Third Order	Fourth Order
Frequencies (Hz)	2107.8	2353.9	2905.1	3456.1

## Data Availability

Not applicable.
